# Health administrative data enrichment using cohort information: Comparative evaluation of methods by simulation and application to real data

**DOI:** 10.1371/journal.pone.0211118

**Published:** 2019-01-31

**Authors:** Bernard C. Silenou, Marta Avalos, Catherine Helmer, Claudine Berr, Antoine Pariente, Helene Jacqmin-Gadda

**Affiliations:** 1 Univ. Bordeaux, INSERM, ISPED, Bordeaux Population Health Center, Bordeaux, France; 2 Inria SISTM Team, Talence, France; 3 Univ. Montpellier, INSERM, Montpellier Cedex, France; 4 CHU de Bordeaux, Pole de Santé Publique, Service de Pharmacologie Médicale, Bordeaux, France; Vrije Universiteit Amsterdam, NETHERLANDS

## Abstract

**Background:**

Studies using health administrative databases (HAD) may lead to biased results since information on potential confounders is often missing. Methods that integrate confounder data from cohort studies, such as multivariate imputation by chained equations (MICE) and two-stage calibration (TSC), aim to reduce confounding bias. We provide new insights into their behavior under different deviations from representativeness of the cohort.

**Methods:**

We conducted an extensive simulation study to assess the performance of these two methods under different deviations from representativeness of the cohort. We illustrate these approaches by studying the association between benzodiazepine use and fractures in the elderly using the general sample of French health insurance beneficiaries (EGB) as main database and two French cohorts (Paquid and 3C) as validation samples.

**Results:**

When the cohort was representative from the same population as the HAD, the two methods are unbiased. TSC was more efficient and faster but its variance could be slightly underestimated when confounders were non-Gaussian. If the cohort was a subsample of the HAD (internal validation) with the probability of the subject being included in the cohort depending on both exposure and outcome, MICE was unbiased while TSC was biased. The two methods appeared biased when the inclusion probability in the cohort depended on unobserved confounders.

**Conclusion:**

When choosing the most appropriate method, epidemiologists should consider the origin of the cohort (internal or external validation) as well as the (anticipated or observed) selection biases of the validation sample.

## Introduction

Health administrative databases (HAD) are a valuable source of data for studying the association between treatment and disease outcome [1–4]. In France, the national inter-regime information system on health insurance (SNIIRAM) database covers the entire French population (65 million inhabitants) [2–4]. This database includes demographic (age, gender, city of residence) and out-hospital reimbursement (drug dispensing and long-term diseases). A 1/97th random permanent sample of SNIIRAM, the general sample of French health insurance beneficiaries (Echantillon Généraliste des Bénéficiaires, EGB), which is representative of the national population of health insurance beneficiaries, was composed in 2005 to allow a 20-year follow-up. These administrative databases are readily available for epidemiological research. The large number of patients without loss of follow-up allows for sufficient powering of studies. Furthermore, the information is plentiful, comprehensive and detailed, without any exclusions [2–4].

Administrative databases are not without limitations. Information on potential confounders is often missing and recent reviews have described current strategies to control for unmeasured confounding in HAD [5–7]. Sensitivity analyses [8–10] have been helpful in the past to adjust for bias due to unmeasured confounders, but they are limited because they cannot control for multiple unmeasured confounding variables. Occasionally, detailed confounding information missing from the HAD (main sample) can be procured from a validation sample that may be a subsample of the HAD (internal validation sample) or another cohort assumed to be representative of the same population (external validation sample). Methods have been developed to incorporate information from the validation sample in the analysis of HAD to reduce confounding bias. Three of these methods are based on the propensity score [11]. Two-stage calibration (TSC) [12] and propensity score calibration (PSC) [13] aim to adjust for the propensity score instead of individual confounders and consider the propensity score computed only with the observed confounders (crude propensity score) as a measure with error of the propensity score including all the confounders (precise propensity score). McCandless and colleagues [14] summarized unobserved confounders in a summary score built using the propensity score methodology and proposed a Bayesian approach (BayesPS) to adjust for this missing score. Multivariate imputation by chained equations (MICE) seeks to adjust directly for the unobserved confounders [15]. MICE has proved to be an effective technique in controlling bias due to unmeasured confounding [16–18]. Unlike the other methods, PSC does not need the outcome variable to be measured in the validation data but relies on the additional surrogacy assumption that measurement error is independent of the outcome variable, given the precise propensity score and exposure [13]. BayesPS does not need the surrogacy assumption but requires either the assumption that the observed confounders are independent of the unobserved ones or a Gaussian linear model for the summary score given the observed confounders; this may be unrealistic for few categorical unmeasured confounders. Simulation studies have shown that PSC is more biased and generally has larger variance than the other three methods, while the performance of BayesPS is similar to that of MICE [12,14]. Several studies have demonstrated the utility of these methods in real applications [8,19–21] but have highlighted the need for additional research on method diagnostics in practical situations to decide which method to use.

The objective of the present study was to compare through simulations the performance of these methods whose required assumptions are more compatible with our target application: large-scale population-based observational HAD and validation cohorts with measures of the outcome variable. Bias and robustness of MICE and TSC were assessed (1) when the validation sample was representative of the same population as the HAD and under different departures from representativeness of (2) the external and (3) the internal validation samples. Our parameter of interest was the log-odds ratio (log(OR)) for the effect of exposure conditional on all the confounders (observed and unobserved).

To illustrate the advantages and limitations of these methods to account for unobserved confounders in HAD, MICE and TSC were then applied to the general sample of EGB to study the relationship between benzodiazepine (BZD) use and fractures in the elderly using two different cohorts, Paquid and Three-City (3C), as validation samples [22–24].

## Materials and methods

### Study population

#### Main sample

The main database was EGB [2]. The data consisted of 60,243 subjects of at least 69 years of age in 2006 who were alive and had not dropped out of the EGB before 2009. BZD users were defined as subjects who had at least one reimbursement for BZD between October 1 and December 31, 2006. We identified 15,638 BZD users while the remaining 44,605 were considered as unexposed. The outcome of interest was fractures of all types arising in the three years following the measure of exposure, that is, between January 1, 2007 and December 31, 2009. Using information on hospital diagnoses, we found 3,260 subjects with at least one fracture. The observed potential confounders were age, gender, exposure to antihypertensive and non-BZD psychotropic medications.

#### Validation samples

The Paquid project, initiated in 1988, was designed to study the risk factors of age-related health conditions [23]. The cohort includes 3,777 subjects of at least 65 years old, from two French departments. Subjects randomly selected from the electoral rolls who agreed to participate were interviewed at their homes by trained neuropsychologists at baseline and subsequently every two or three years. At each visit, information on fractures since the last visit and drugs used at the time of the visit was collected. The validation sample consisted of 1,342 subjects visited in the 5th (T5, in 1993–94) and 8th (T8, in 1996–97) follow-up years with complete data regarding drugs used and confounding factors at T5 and fractures at T8. This ensured that the validation sample was as close as possible to the EGB sample while optimizing the sample size. At T5, information on potential confounders available in EGB (age, gender, antihypertensive and non-BZD psychotropic drugs used) was collected as well as information on potential confounders missing from EGB: body mass index (BMI), educational level (primary school diploma denoted high education versus no diploma or no education denoted low education) and depressive symptomatology measured by the Center for Epidemiologic Studies Depression Scale (CESD) as a binary covariate (subjects were considered as depressed if they obtained a score of more than 17 for males and 23 for females out of 60 on the CESD scale).

The 3C study is a population-based longitudinal study of the relation between vascular diseases and dementia including 9,294 participants aged 65 years and older at baseline in 1999 and living in three French cities (Bordeaux, Dijon and Montpellier) [24]. The validation sample for this analysis consisted of 2,231 subjects from Bordeaux and Montpellier visited in the 4th (T4 in 2003–04) and 7th (T7 in 2006–07) years of follow-up. Exposure to medications and confounders were collected at T4 and fractures at T7, as in Paquid.

### Ethics statement

INSERM, as a health research institute, has been authorized to use the EGB database by the French data protection authority (Commission Nationale de l’Informatique et des Libertés, CNIL), provided that the researcher follows specific training with certification, as the first and fifth authors (Bernard Silenou, Antoine Pariente) have obtained.

The Ethics Committee of Kremlin-Bicêtre University Hospital and Bordeaux University Hospital respectively approved study protocols for the 3C and Paquid cohorts, and each participant signed a written informed consent. All data were fully anonymized.

### Adjustment methods for unobserved confounders

Let *Y* be the binary outcome and *X* the binary exposure variable. Let ***C*** be the vector of confounders measured in both the main and the validation data and ***U*** the vector of confounders measured only in the validation data.

#### Two-stage calibration

The crude propensity score is defined as the probability of being exposed given observed confounders ***C*** (*PS*_*C*_ *= P(X = 1|****C***)) and the precise propensity score as the probability of being exposed given confounders ***C*** and ***U*** (*PS*_*P*_ *= P(X = 1|****C***,***U***)) [13,25]. *PS*_*C*_ and *PS*_*P*_ are estimated by logistic regression in the pooled (main+validation) and validation data, respectively.

Lin and Chen [12] defined two models for the outcome adjusting either for *PS*_*C*_ or *PS*_*P*_

$$logit[P(Y=1|X,C)]=\delta +\gamma X+\theta f(PS_{C})$$ (1)

$$logit[P(Y=1|X,C,U)]=\alpha +\beta X+\varphi g(PS_{P})$$ (2)

where *f* and *g* are identity or suitable transformation functions, e.g. spline functions. TSC aims to estimate *β* in the pooled sample ($\bar{\beta }$) from $\hat{\beta }$ and $\hat{\gamma }$ estimated in the validation sample, and $\bar{\gamma }$ estimated in the pooled sample using [12]

$$\bar{\beta }=\hat{\beta }-\frac{\lambda }{\nu }\;(\hat{\gamma }-\bar{\gamma })$$

and $var(\bar{\beta })=var(\hat{\beta })-\frac{\lambda^{2}}{\nu }$ where *λ* is the covariance between $\hat{\beta }$ and $\left(\hat{\gamma }-\bar{\gamma }\right)$ and *ν* is the variance of $\left(\hat{\gamma }-\bar{\gamma }\right)$; *λ* and *ν* are estimated by the sandwich estimator as detailed in the web appendix of [12]. Like PSC and BayesPS, TSC requires that the propensity score models are well specified and that the validation sample is representative of the main sample. More precisely, $\hat{\gamma }$ and $\bar{\gamma }$ are assumed to be unbiased estimates of γ and $\hat{\beta }$ is assumed to be an unbiased estimate of *β*. We will see later that departures from representativeness that do not invalidate the above assumptions are permitted (for instance, different marginal distributions of either C, X or Y). On the other hand, TSC does not need assumption regarding the relationship between *PS*_*C*_ and *PS*_*P*_.

#### Multiple imputation

Unobserved confounders in the main sample can be considered as missing data in the pooled sample, and multiple imputation such as MICE may be used to adjust for these unobserved confounders [15]. However, in this context where the proportion of missing observations for ***U*** is vast, it is recommended to increase the number of imputations [26]. The multiple imputation approach requires the missing-at-random assumption, i.e. that the observation probability for U, which is the probability of belonging to the validation cohort in this context, does not depend on U. Moreover, parametric assumptions are needed for imputation models. We used logistic regression as an imputation model for binary variables and predictive mean matching, which is a robust method for non-Gaussian variables, for quantitative variables [27]. All variables were included in each imputation model to preserve the correlation structure in the data.

### Simulation study

We compared the performances of the methods when the external validation sample is representative of the same population as the main sample and under different departures from representativeness. The validation data were considered to be representative when the multivariate distribution of all the variables (*Y*, *X*, ***C***, ***U***) in the validation sample is identical to that of the main sample. We considered two observed (***C*** = (*C*1, *C*2)) and two unobserved (***U*** = (*U*1, *U*2)) confounders. The binary exposure (*X*) and the binary outcome (*Y*) were generated using the following logistic models:

$$logit(P(X=1|C,U))=\lambda_{0}+\lambda_{1}C_{1}+\lambda_{2}C_{2}+\lambda_{3}U_{1}+\lambda_{4}U_{2}$$ (3)

$$logit(P(Y=1|X,C,U))=\beta_{0}+\beta X+\beta_{1}C_{1}+\beta_{2}C_{2}+\beta_{3}U_{1}+\beta_{4}U_{2}$$ (4)

All the data were generated under the assumption of no exposure effect (*β = 0)*, thus avoiding the issue of non-collapsibility of the OR [28]. Thus, a difference in parameter estimates of *β* with or without conditioning on unmeasured confounders *U*_1_ and *U*_2_ would solely be due to the confounding bias of these unmeasured confounders.

The simulation proceeded by generating a population of n_P_ = 21,000 subjects. In scenarios 1 and 2, we focused on external validation samples, while in scenario 3, we generated an internal validation sample. For scenarios 1 and 2, a representative main sample (n_M_ = 10,000) was randomly drawn from the population while the validation sample (n_V_ = 1,000) was extracted from the remaining 11,000 subjects. In scenario 1 the validation sample was representative of the population, while in scenario 2 it was not. The variable ***U*** in the main data was considered as missing. For scenario 1, two series of simulations were run by varying the distribution of the confounders:

***Scenario 1*.*a***: Confounders ***U*** and ***C*** were generated from a standard normal distribution.

***Scenario 1*.*b***: Confounders ***U*** and ***C*** were generated from non-Gaussian distributions roughly mimicking the distributions of the sex (*C*_1_), age (*C*_2_), CESD (*U*_1_) and education (*U*_2_) variables in the Paquid cohort. *C*_1_ and *U*_2_ were binary while *C*_2_ and *U*_1_ followed truncated log normal distributions. Additional simulations were performed with five unobserved confounders for scenarios 1.a and 1.b (for 1.b U_3_ was binary, U_4_ was truncated log-normal and U_5_ was Gaussian).

***Scenario 2***: The population was generated as in scenario 1.a and non-representative validation samples were selected to investigate the sensitivity of each method to various selection biases. The probability of inclusion in the validation sample was a function of either *X*, *Y*, *C* or C * Y (in ***Scenario 2*.*a***), where * represents an interaction effect between two variables, *X + Y* or X*Y (in ***Scenario 2*.*b***), or *U*, U * X or U * Y (in ***Scenario 2*.*c***). Scenarios 2.a and 2.b correspond to missing-at-random mechanisms, hence MICE is expected to be robust, while for scenario 2.c, data are missing not at random and MICE is expected to fail. TSC requires that the association between *Y* and *X* given ***C*** alone and given ***C*** and ***U*** be identical in the validation and the main sample. This assumption was violated in scenarios 2.b and 2.c.

***Scenario 3*:** We performed an additional set of simulations with a non-representative internal validation sample with inclusion probability depending on X * Y (similar to scenario 2b).

**Fig 1:**
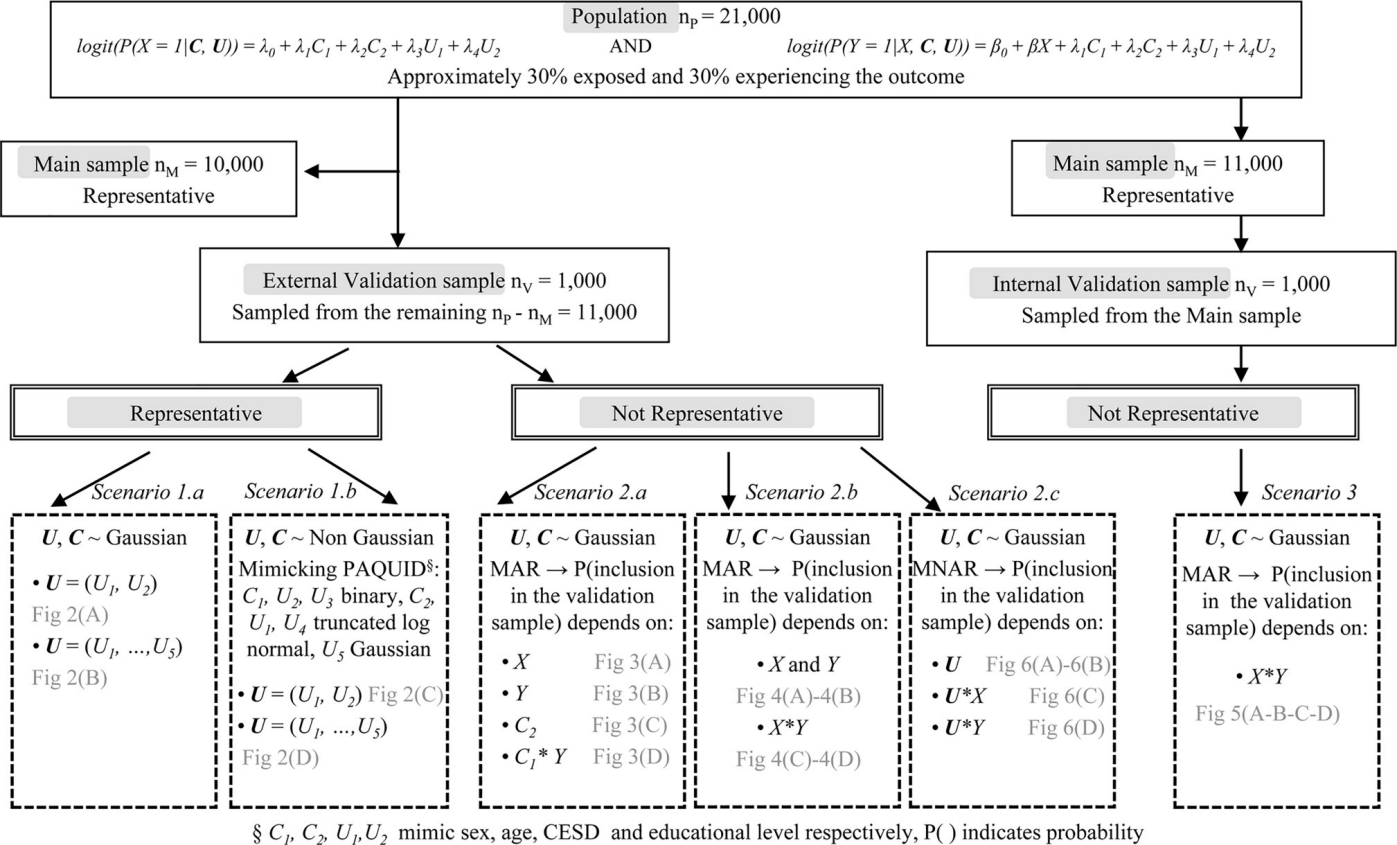
Flowchart of the data generation procedure by scenario for internal and external validation samples. MAR and MNAR correspond to missing-at-random and missing-not-at-random mechanisms, respectively.

Fig 1 shows the flowchart of the data generation procedure by scenario. For each scenario, we generated 500 data sets. Parameter values for data generation are specified in S1 File. We compared estimates of the log(OR) for the exposure X obtained from MICE with 10 imputations, TSC with either identity function (TSC) or natural cubic spline using 2 knots (TSC_SP) for *f* and *g* in models (1) and (2), logistic regression on the main data adjusting for (***U***,***C***) (UC_MAIN) or for ***C*** only (C_MAIN), on the pooled data adjusting for (***U***,***C***) (UC_POOL) or for ***C*** only (C_POOL) and on the validation data adjusting for (***U***,***C***) (UC_VAL).

The bias (with respect to the target parameter β = 0) was computed as the mean of the estimates $\bar{\hat{\beta }}$ over the 500 replicates. The efficiency of the various estimates was compared through the cross-replications standard error (empirical standard error, $ESE(\hat{\beta })$). For each data set (k = 1,…,500), the 95% confidence interval of the estimate was computed as ${\hat{\beta }}_{k}\pm 1.96\;ASE({\hat{\beta }}_{k})$ where $ASE({\hat{\beta }}_{k})$ is the estimated asymptotic standard error of the considered estimate on sample k. The coverage rate of the confidence interval was computed as the proportion of times this CI included the true value 0 over the 500 replicates. A coverage rate will be close to the nominal value of 95% means if (i) the bias for the parameter estimate $\hat{\beta }$ is negligible compared to its variance and (ii) the variance of $\hat{\beta }$ is correctly estimated. With 500 replicates, the coverage rate is significantly different from 95.0 when it is outside 93.1–96.9. Finally the mean square error (MSE) was computed as $MSE=\sum_{k=1}^{500}(\beta -{\hat{\beta }}_{k}{)}^{2}/500$; the MSE allows a global comparison of the estimators since it is the sum of their square bias and their variance. Analyses were performed with R version 3.2.3.

## Results

### Simulation

**Fig 2:**
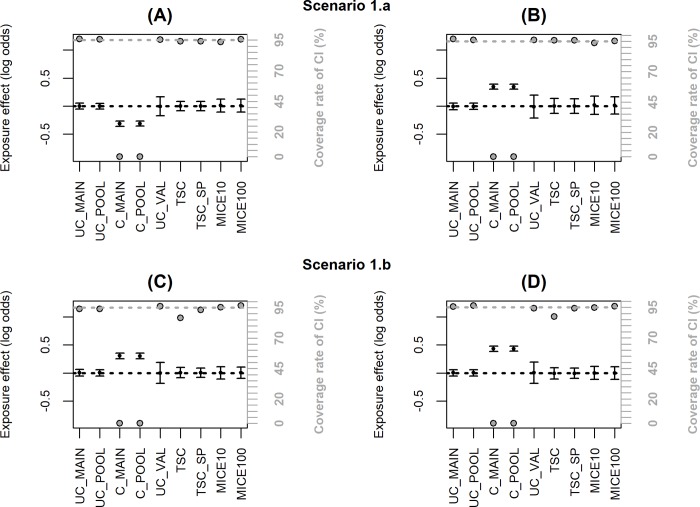
**Simulation results with a representative external validation sample (scenario 1); coverage rate of 95% confidence interval (grey dot with black margin) and mean estimated log-odds ratio for the exposure effect (black dot) ± empirical standard error:** (A) two unobserved Gaussian confounders, (B) five unobserved Gaussian confounders, (C) two unobserved non-Gaussian confounders, (D) five unobserved non-Gaussian confounders. The grey dotted line corresponds to the nominal value of the coverage rate of the 95% confidence interval. The black dotted line is the true value of β (0).

**Fig 3:**
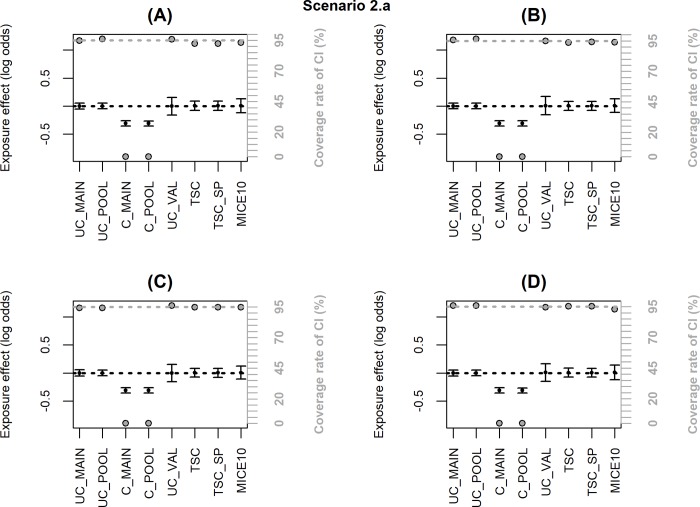
Simulation results when the inclusion probability in the external validation sample (notated P) depends only on *X*, *Y*, *C* or C*Y (scenario 2.a); coverage rate of 95% confidence interval (grey dot with black margin) and mean estimated log-odds ratio for the exposure effect (black dot) ± empirical standard error: (A) logit(P) = −2.7+log(4)*X*, (B) logit(P) = −2.7+log(4)*Y*, (C) logit(P) = −2.7+log(4)*C*_*2*_, (D) logit(P) = −2.5+log(2)*C*_*1*_+log(2)*Y*+log(4)*C*_1_*Y. The grey dotted line corresponds to the nominal value of the coverage rate of the 95% confidence interval. The black dotted line is the true value of β (0).

**Fig 4:**
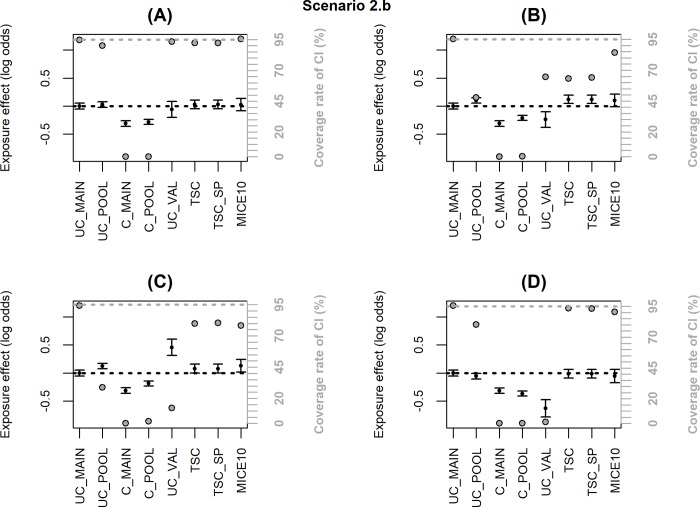
Simulation results when the inclusion probability in the external validation sample (notated P) depends on *X + Y* or X*Y (scenario 2.b); coverage rate of 95% confidence interval (grey dot with black margin) and mean estimated log-odds ratio for the exposure effect (black dot) ± empirical standard error: (A) logit(P) = -2.6+log(2)*X*+log(2)*Y*, (B) logit(P) = -3.2+log(4)*X*+log(4)*Y*, (C) logit(P) = -2.7+log(2)*X*+log(2)*Y*+log(2)X*Y, (D) logit(P) = -2.5+log(2)*X*+log(2)*Y*-log(2)X*Y. The grey dotted line corresponds to the nominal value of the coverage rate of the 95% confidence interval. The black dotted line is the true value of β (0).

**Fig 5:**
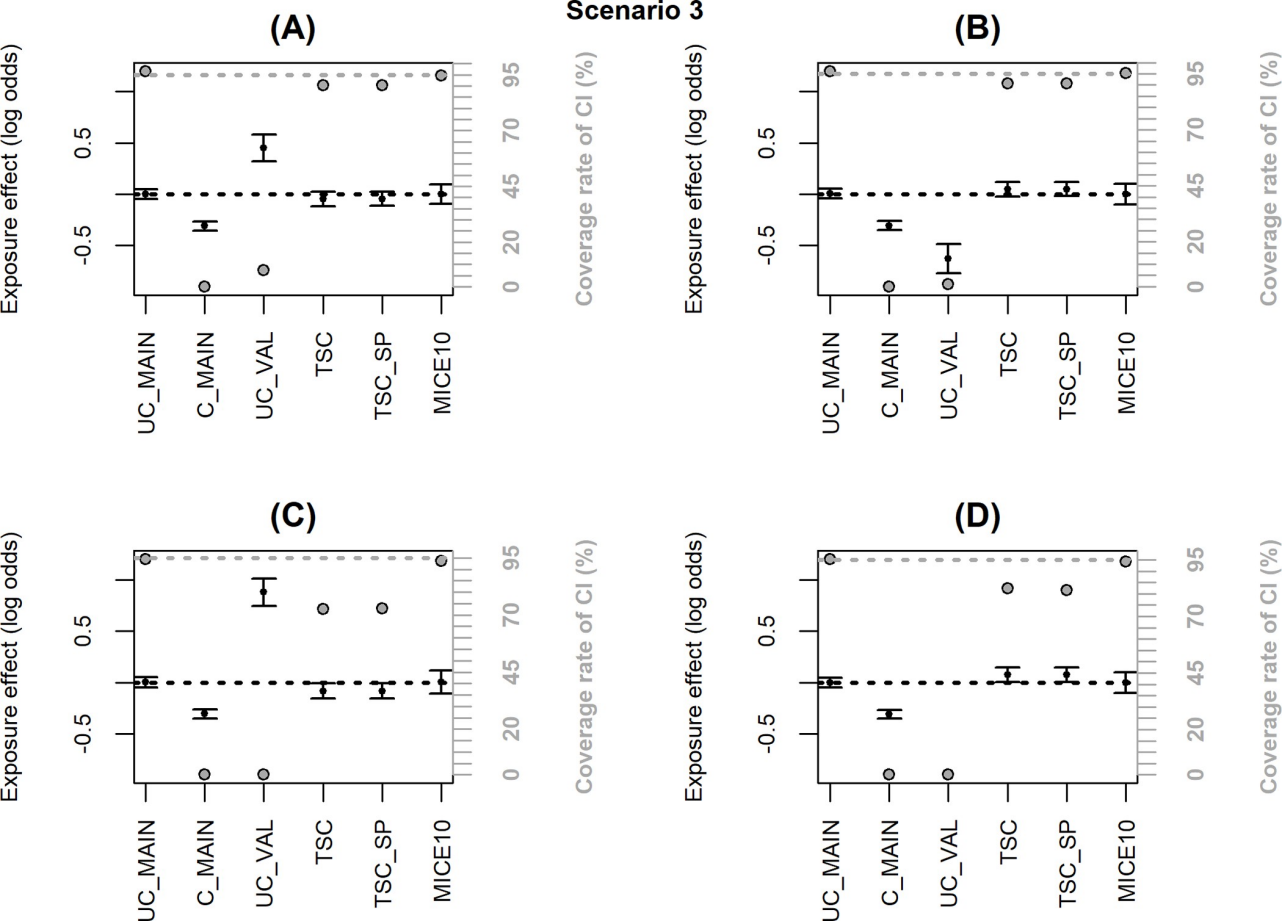
Simulation results with a non-representative internal validation sample where the inclusion probability (notated P) depended on X*Y (scenario 3); coverage rate of 95% confidence interval (grey dot with black margin) and estimated log-odds ratio for the exposure effect (black dot) ± empirical standard error: (A) logit(P) = −2.7+log2X+log2Y+log2X*Y, (B) logit(P) = −2.5+log2X+log2Y−log2X*Y, (C) logit(P) = −2.8+log2X+log2Y+log4X*Y, (D) logit(P) = −2.4+log2X+log2Y−log4X*Y. The grey dotted line corresponds to the nominal value of the coverage rate of the 95% confidence interval. The black dotted line is the true value of β (0).

**Fig 6:**
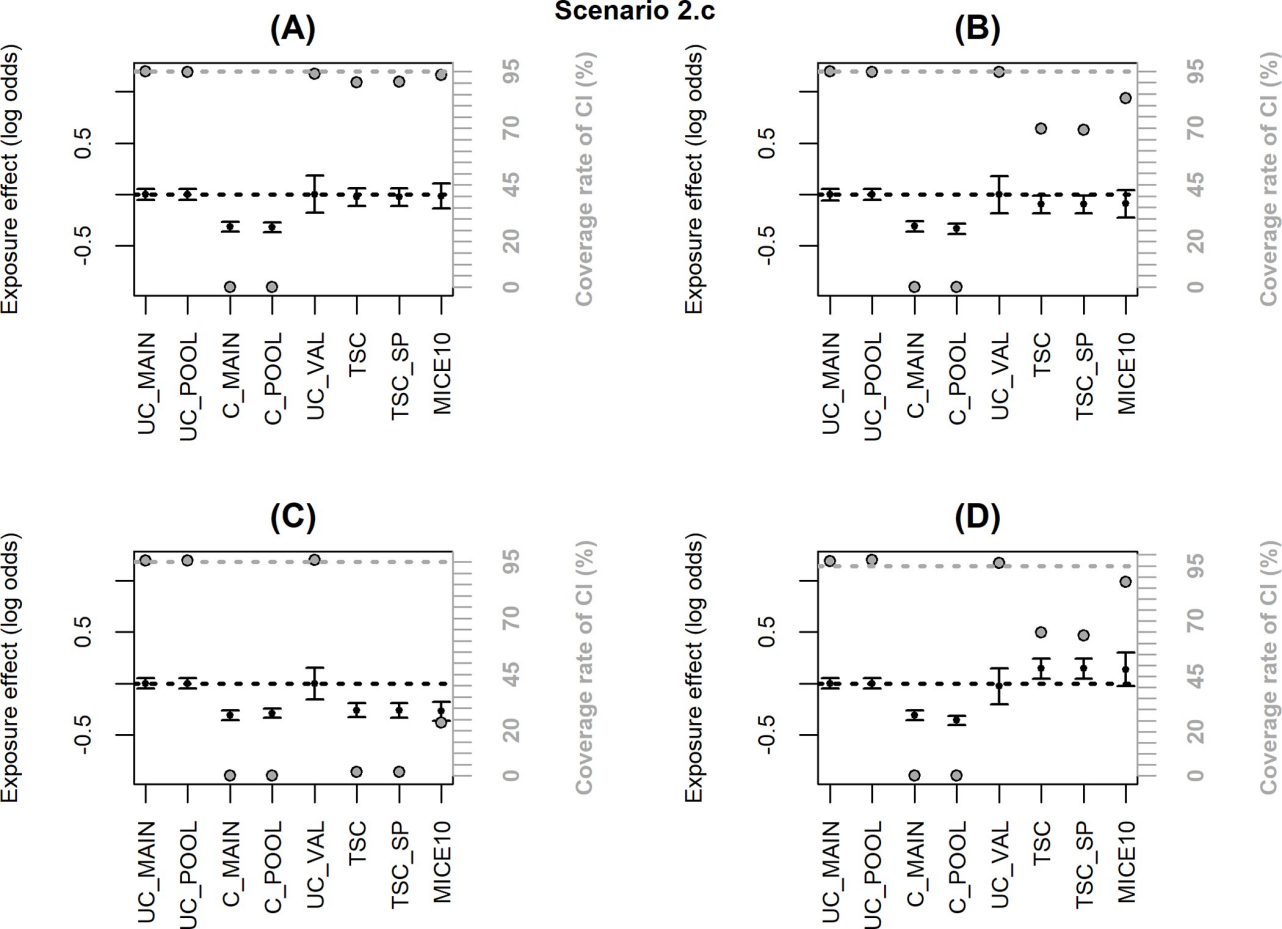
**Simulation results when the inclusion probability in the external validation sample (notated P) depends on *U*_1_; coverage rate of 95% confidence interval (grey dot with black margin) and mean estimated log-odds ratio for the exposure effect (black dot) ± empirical standard error:** (A) logit(P) = -2.2+log(2)*U*_*1*_, (B) logit(P) = -2.7+log(4)*U*_*1*_, (C) logit(P) = -2.3+log(2)*U*_*1*_+log(2)X*U_*1*_, (D) logit(P) = -2.5+log(2)*U*_*1*_+log(2)Y*U_*1*_. The grey dotted line corresponds to the nominal value of the coverage rate of the 95% confidence interval. The black dotted line is the true value of β (0).

Figs 2–6 summarize the main simulation results through the mean estimates ± ESE and the coverage rates of the 95% confidence interval based on the asymptotic standard error estimated on each data set. S1–S5Tables display detailed simulation results including mean asymptotic standard error, empirical standard error, mean square error and mean computation time.

Fig 2 and S1 Table display the results for the estimates of the effect of exposure when the validation data are representative of the same population as the main sample (scenario 1). The estimate adjusted only for ***C*** from the main sample (C_MAIN) highlights the bias due to the unmeasured confounders. The adjusted estimate from the validation sample (UC_VAL) is unbiased but has a larger standard error owing to the small sample size. All the correction methods are unbiased with a coverage rate of the CI close to 95%, and the two TSC estimators appear to be the most efficient since their ESE are the smallest. This is confirmed by comparing the MSE which is the sum of the bias and variance: TSC and TSC_SP have the smallest MSE in S1 Table. However, Fig 2C and 2D show a slight under-coverage of the CI for TSC when the confounders are non-Gaussian (86.6% and 87.4% instead of 95%). This is explained by the slight underestimation of the standard error (ASE compared to ESE in case 1.b in S1 Table) because the variance of estimated parameters from the propensity score model is neglected [29]. By using the true parameter value for the propensity score model instead of the estimates obtained on the validation sample, this underestimation disappears (results not shown). When additional spline parameters are estimated in TSC_SP, the impact of the variance of the propensity score model is negligible and the coverage rate of the CI remains correct. In scenario 1, we also compared MICE using either 10 or 100 imputations. Results in S1 Table show negligible differences between 10 and 100 imputations in term of bias and efficiency but a 10-fold increase in computation time. For the other scenarios, MICE was thus computed using 10 imputations only. The TSC approaches requires the least computation time: less than 0.1seconds per sample for 5 unobserved confounders versus 30 or 40 seconds for MICE with 10 imputations depending on the imputation methods (predictive mean matching takes more time than logistic regression).

When the inclusion probability in the validation sample depends only on *X*, *Y*, ***C*** or C*Y, the associations between *X* and *Y* given ***C*** alone and given ***C*** and ***U*** are identical in the main and the validation samples and U is missing at random. Thus, both TSC and MICE remain unbiased with a coverage rate close to the nominal value (scenario 2.a, Fig 3, S2 Table).

When the inclusion probability in the external validation sample depends on *X* and *Y*, TSC and MICE lead to significant bias (scenario 2.b, Fig 4 and S3 Table). When the dependence on *X* and *Y* is moderate (OR = 2, Fig 4A), the bias has negligible impact on the coverage rate of the 95% CI; but the bias increases with the strength of the dependence (OR = 4, Fig 4B) and more dramatically when the inclusion probability depends on an interaction X*Y (Fig 4C and 4D) leading to a major undercoverage of the CI. These bad results were expected for TSC since the selection makes the association between *Y* and *X* different between the validation and the main sample (as shown by the bias in UC_VAL). However, bias in MICE estimates may appear surprising because the missing data are at random (MAR). To explain this result, we must emphasize that the objective of the correction methods is to estimate the adjusted association between *X* and *Y* in the population of which the pooled sample is representative. When a non-representative external validation sample is used, the pooled and main samples do not reflect the same population and the association between *X* and *Y* may be different in the two populations. Indeed, in Fig 4 and S3 Table, UC_MAIN and UC_POOL are different and we can see that MICE estimate is close to UC_POOL. On the other hand, when an internal validation sample is used (i.e. the validation sample is a subsample of the main HAD), the pooled and main samples are identical even if the validation sample is not representative. In scenario 3, we generated a non-representative internal validation sample where the inclusion probability depended on X*Y and we checked that MICE was unbiased with nominal coverage rate while TSC was still biased (Fig 5 and S4 Table).

Finally, all the correction methods fail when the inclusion probability in the validation sample depends on ***U*** (scenario 2.c, Fig 6, S5 Table), and especially when it depends on X*U or Y*U (Fig 6C and 6D). The imputation method fails because the confounders are not missing at random, while TSC fails because the confounding effect of ***U*** is different in the validation and the main sample. Note however that, adjusting on *U*, the parameter estimate in the validation sample is unbiased (UC_VAL) with this scenario (thus UC_POOL is also unbiased).

### Application

Table 1 presents the distribution of all the variables in the main and validation samples. BZD users tended to be less educated and more depressed, suggesting that these factors could be confounders. We observed some differences in the distributions of BZD users (X), fractures (Y) and educational levels (U) between the three samples.

**Table 1:** Description of study population in main (EGB, n = 60,243, 2006–2009) and validation samples (Paquid, n = 1,342, 1993–1997; 3C, n = 2,231, 2003–2007) in France.

Baseline variables	EGB		Paquid		3C	
	No-BZD	BZD	No-BZD	BZD	No-BZD	BZD
**n (%)**	44,605 (74)	15,638 (26)	907 (68)	435 (32)	1,727 (77)	504 (23)
**Age in y, mean (SD)**	78.1 (7.2)	78.1 (6.4)	77.9 (5.7)	78.1 (5.2)	76.6 (4.8)	77.8 (5.1)
**Fractures %**	4	8	8	11	10	12
**Female %**	57	73	51	77	59	75
**Anti-hypertensive %**	47	72	58	70	61	61
**Other psychotropics %**	8	32	10	29	9	29
**High education %**			77	68	94	90
**CESD %**			5	18	7	16
**BMI in kg/m**^**2**^**, mean (SD)**			25.0 (3.7)	24.4 (3.8)	25.5 (3.8)	25.3 (4.7)

Abbreviations: BMI, body mass index; BZD, exposure to benzodiazepine; CESD, Center for Epidemiologic Studies Depression Scale.

MICE and TSC were applied to estimate the log(OR) of the association between exposure to BZD and fractures adjusted for the observed confounders ***C*** (age, gender, anti-hypertensive and non-BZD psychotropic) and unobserved confounders ***U*** (BMI, CESD and education). As a comparison, we also estimated the log(OR) adjusted for ***C*** in EGB (C_MAIN) and for (***U*, *C***) in the validation sample (UC_VAL). Results are displayed in Table 2. Without adjusting for ***U*** in EGB, BZD users had a higher risk of fractures (log(OR) = 0.36, 95% CI: 0.28, 0.44). By adjusting for BMI, education and CESD in the Paquid cohort, the regression parameter dropped by approximately half (0.17, [-0.25, 0.59]), while it was null in 3C. The three correction methods using Paquid as validation sample highlighted an association between BZD and fractures in the pooled sample, with log(OR) close to the adjusted estimate in the validation sample but with smaller variance. These results were consistent with the simulation results. On the other hand, although the estimated adjusted log(OR) was null in 3C (UC_VAL), estimations obtained with the correction methods were very close to the estimate adjusted only for ***C*** in EGB.

**Table 2:** Estimates of exposure effect (log-odds ratio) of association between BZD and fractures; EGB database (n = 60,243, 2006–2009) and Paquid (n = 1,342, 1993–1997) and 3C (n = 2,231, 2003–2007) cohorts in France.

Methods	log(OR)	SE	95% CI
**C_MAIN**	0.36	0.04	0.28, 0.44
**EGB and Paquid**			
**UC_VAL**	0.17	0.21	-0.25, 0.59
**TSC**	0.20	0.05	0.11, 0.29
**TSC_SP**	0.23	0.04	0.14, 0.31
**MICE**	0.25	0.07	0.12, 0.38
**EGB and 3C**			
**UC_VAL**	0.00	0.17	-0.33, 0.33
**TSC**	0.32	0.02	0.28, 0.37
**TSC_SP**	0.34	0.02	0.30, 0.37
**MICE**	0.32	0.04	0.24, 0.40

Abbreviations: CI, confidence interval; OR, odds ratio; SE, standard error. MICE was implemented with 100 imputations. TSC_SP was implemented with 5 knots.

To explain these differential results, we estimated two logistic regressions to identify factors associated with inclusion in 3C or Paquid, respectively, versus EGB. The occurrence of fractures and exposure to BZD were both associated with inclusion in Paquid (OR = 1.79, p<0.001 and OR = 1.31, p<0.001, respectively) but their interaction was not significant (OR = 0.83, p = 0.35). According to our simulations (Fig 4A), the bias of adjustment methods in this case should be negligible. On the other hand, the interaction between the occurrence of fractures and exposure to BZD was significant in the logistic regression for inclusion in 3C versus EGB (OR = 0.64, p = 0.007). This means that 3C sample is not representative of the EGB population with respect to the association between BZD and fractures. This sample is an urban and highly educated sample where the causes of fractures or the BZD use pattern may be different from the overall French population. This corresponds to a situation where the correction methods are highly biased (Fig 4C and 4D).

## Discussion

Claims data are increasingly used for epidemiological research, but results may be biased since information on confounders is missing. A key approach to this problem is to include confounder data from cohort studies in the same population. Strategies to control for unmeasured confounding in HAD have been the subject of recent surveys [5–7]. The surveys provide general recommendations on the choice of the strategy depending, for example, on the study design or the existence or not of a validation sample. However, to our knowledge, no study to date has provided recommendations on how to choose methods that include confounder data from cohort studies in the event of a lack of representativeness of the cohort and depending on the nature of the cohort (internal or external).

Our findings show that estimates from TSC and MICE are unbiased when the validation sample is representative of the population covered by the HAD. Multiple imputation works well in this framework despite the very high rate of missing information on confounders, even in cases where the unobserved confounders have nonstandard distributions, thanks to the robustness of imputation by predictive mean matching [27]. Nevertheless, MICE requires more computation time—an issue to consider when dealing with very large HAD—and is less efficient than TSC. When unobserved confounders have nonstandard distributions, variances may be slightly underestimated with TSC, but TSC_SP is more robust. A way to avoid this issue could be to apply TSC, adjusting directly on C and U instead of the propensity scores.

All methods are robust to non-representativeness except when the validation and main samples differ in the distribution of unobserved confounders or the distributions of both the outcome and the exposure. In the former case, which is untestable in practice, all methods are biased while in the latter, MICE provides an unbiased estimate in the pooled sample. Interestingly, the latter assumption can be evaluated in practice, as was illustrated in the BZD-fracture study.

We focused mainly on external validation samples because this is the most frequent situation when existing cohorts are used as validation samples. Owing to differences in time periods, selection procedures and participation rates, such cohorts are not expected to be completely representative of the population targeted by the HAD. This motivated the investigation of the impact of departures from representativeness. However, some nationwide HAD are almost exhaustive, so existing cohorts in the country may be considered as internal validation samples if a linkage between the databases is possible. A clear advantage of internal validation is that the measure of the exposure, outcome and observed confounders are common in both samples. In this context, the robustness of MICE is useful when the validation and main samples differ according to the distributions of both the outcome and exposure.

The analysis of the relationship between BZD and fractures using EGB data illustrates how these methods may be applied and, to some extent, how their validity may be evaluated in real data analyses. While this is not the best design for this study because it may suffer from a survival bias, the analysis confirms that elderly users of BZD have an elevated risk of experiencing a fracture compared to unexposed subjects after controlling for confounders including BMI, education and CESD. The measures of exposure to drugs and outcome differed between the validation and main samples. In Paquid and 3C, drug use was self-reported, but these measures can be considered reliable as the interviewer checked medication containers. They include over-the-counter drugs that are not collected in EGB, which records drug delivery from pharmacies based on prescriptions. However, antihypertensives, BZD and most non-BZD psychotropics cannot be bought in France without a prescription. Moreover, fractures in EGB are based on clinical diagnosis in hospitals, while fractures in the previous 3-year period were self-reported in the validation sample. Memory bias and diagnosis error are possible but probably low for a traumatic event such as a fracture.

Two important points should be made about this study. First, we compared estimates of the effect of exposure adjusting either for individual confounders or for propensity scores. However, we checked both in the simulation study and in the application that the differences between these estimates were negligible (results not shown). Second, in general, OR is a non-collapsible measure [29], meaning that conditional and marginal ORs may be different even without a confounding effect. In the application, the differences between ORs adjusted for ***U*** and ***C*** and adjusted only for ***C*** may be due to both a confounding effect and non-collapsibility. Nevertheless, the simulations were conducted under the assumption of no exposure effect, where the OR is collapsible.

In conclusion, TSC and MICE can efficiently reduce confounding bias from unobserved confounders in large-scale studies when a validation sample with complete information on confounders is available. The origin (internal or external) of the validation sample as well as the anticipated or observed selection biases must be considered when choosing the most appropriate method. Future work could aim at improving variance estimates in TSC by accounting for the estimation of propensity score [29], or at correcting for selection bias in the validation sample through weighting approaches.

## Supporting information

S1 File Complements to simulation design. (DOCX)

S1 Table Simulation results for scenario 1. (DOCX)

S2 Table Simulation results for scenario 2.a. (DOCX)

S3 Table Simulation results for scenario 2.b. (DOCX)

S4 Table Simulation results for scenario 3. (DOCX)

S5 Table Simulation results for scenario 2.c. (DOCX)
