# Supplementary material for: Health administrative data enrichment using cohort information: Comparative evaluation of methods by simulation and application to real data
Source: PLoS One. 2019 Jan 31;14(1):e0211118. doi: 10.1371/journal.pone.0211118 (PMC6354983; doi:10.1371/journal.pone.0211118)
Supplement: S1 File — (DOCX) [file pone.0211118.s001.docx]

**S1 File: Complements to simulation design**

**Simulation parameter values of the population**

We considered two observed (***C*** = (*C*1, *C*2)) and two unobserved (***U*** = (*U*1, *U*2)) confounders, a binary exposure (*X*) and a binary outcome (*Y*). Variables *X* and *Y* were generated using a logistic model depending on ***C*** and ***U*** as defined by equations (1[)](#_bookmark5) and (2), respectively:

*logit*(*P*(*X* = 1*|****C,U***)) = *λ*0 + *λ*1*C*1 + *λ*2*C*2 + *λ*3*U*1 + *λ*4*U*2 (1)

*logit*(*P*(*Y* = 1*|X*, ***C,U***)) = *β*0 + *βX* + *β*1*C*1 + *β*2*C*2 + *β*3*U*1 + *β*4*U*2 (2)

***Scenario 1.a****:* Confounders ***U*** and ***C*** were generated from a standard normal distribution and model [(1)](#_bookmark5) and [(2)](#_bookmark6) were used to generate *X* and *Y* respectively with *λ*_0_ = −0.9, *λ*_1_ = 0.1, *λ*_2_ = 0.1, *λ*_3_ = −0.7, *λ*_4_ = 0.5, *β*_0_ = −0.9, *β*_1_ = −0.1, *β*_2_ = 0.3, *β*_3_ = 0.7, *β*_4_ = 0.2. We assumed no exposure effect (*β* = 0).

***Scenario 1.b****:* Confounders ***U*** and ***C*** were generated from non-Gaussian distributions roughly mimicking the variables sex (*C*_1_), age (*C*_2_), CESD (*U*_1_) and PSD (*U*_2_) in the Paquid cohort. *C*_1_ and *U*_2_ were binary with probability *p* = 0.7; *U*_1_ followed a truncated log normal distribution rounded to the closest integer such that min=0, mean=10.5, SD=9.8, max=55; *C*_2_ followed a truncated log normal distribution with min=65, mean=79, SD=10.2 and max=102. The parameters for model [(1)](#_bookmark5) and [(2)](#_bookmark6) were *λ*_0_ = −1.787, *λ*_1_ = 0.619, *λ*_2_ = 0.001, *λ*_3_ = 0.063, *λ*_4_ = −0.190, *β*_0_ = −8.176, *β*_1_ = 0.221, *β*_2_ = 0.096, *β*_3_ = 0.042 and *β*_4_ = −1.841. We assumed no exposure effect (*β* = 0).

***Scenario 2****:* The population was generated as in *Scenario 1.a*.

***Scenario 3****:* The population was generated as in *Scenario 1.a*.

**Simulation design with external validation sample and five Gaussian unobserved confounders**

All confounders were independent Gaussian variables (*C*1, *C*2, *U*1, *U*2, *U*3, *U*4, *U*5 *∼ N*(0, 1)) and equations (3) and (4) were used to generate *X* and *Y* respectively.

*logit*(*P*(*X* = 1|***C,U***)) = *−*0.8 + 0.1*C*_1_ + 0.1*C*_2_ *−* 0.7*U*_1_ + 0.5*U*_2_ + 0.5*U*_3_ + 0.5*U*_4_ + 0.5*U*_5_ (3)

*logit*(*P*(*Y* = 1|*X*,***C,U***)) = *−*1.1 + 0*X −* 0.1*C*_1_ + 0.3*C*_2_ + 0.7*U*_1_ + 0.4*U*_2_ + 0.4*U*_3_ + 0.5*U*_4_ + 0.5*U*_5_ (4)

**Simulation design with external validation sample and five non Gaussian unobserved confounders**

We simulated Bernoulli variables for *C*_1_, *U*_2_ and *U*_3_, ((*C*_1_, *U*_2_)*∼Bern*(*p* = 0.7), *U*_3_*∼Bern*(*p* = 0.5) ) and standard Gaussian for *U*_5_. *U*_1_ followed a truncated log normal distribution rounded to the closest integer such that min=0, mean=10.5, SD=9.8, max=55; *C*_2_ followed a truncated log normal distribution with min=65, mean=79, SD=10.2 and max=102; *U*_4_ followed a truncated log normal distribution with min=0, mean=1.5, SD=1.5 and max=10. *X* and *Y* were generated with models (5) and (6) respectively:

| *logit*(*P*(*X* = 1\| **C, U**)) = *−*2.3 + 0.619*C*_1_ + 0.001*C*_2_ + 0.063*U*_1_ *−* 0.190*U*_2_ + 0.2*U*_3_ + 0.2*U*_4_ +0.3*U*_5_ | (5) |
| --- | --- |
| *logit*(*P*(*Y* = 1\| *X*, **C, U**)) = *−*8.4 + 0*X* + 0.221*C*_1_ + 0.096*C*_2_ + 0.042*U*_1_ *−* 1.841*U*_2_ + 0.2*U*_3_ + 0.2*U*_4_+0.3*U*_5_ | (6) |
