# Supplementary material for: Health administrative data enrichment using cohort information: Comparative evaluation of methods by simulation and application to real data
Source: PLoS One. 2019 Jan 31;14(1):e0211118. doi: 10.1371/journal.pone.0211118 (PMC6354983; doi:10.1371/journal.pone.0211118)
Supplement: S1 Table — (DOCX) [file pone.0211118.s002.docx]

**S1 Table. Simulation results for the estimate of β = log(OR_YX_) when the validation sample is external and representative (Scenario 1)**

|  | **UC_MAIN** | **UC_POOL** | **C_MAIN** | **C_POOL** | **UC_VAL** | **TSC** | **TSC_SP** | **MICE10** | **MICE100** |
| --- | --- | --- | --- | --- | --- | --- | --- | --- | --- |
| **Scenario 1.a: 2U (C,U) ∼ Gaussian** | | | | | | | | | |
| Bias | 0.000 | -0.001 | -0.312 | -0.312 | -0.004 | 0.001 | 0.001 | 0.010 | 0.009 |
| ASE | 0.054 | 0.051 | 0.048 | 0.046 | 0.170 | 0.077 | 0.078 | 0.119 | 0.119 |
| ESE | 0.053 | 0.050 | 0.048 | 0.046 | 0.168 | 0.083 | 0.084 | 0.117 | 0.113 |
| MSE | 0.003 | 0.003 | 0.100 | 0.099 | 0.028 | 0.007 | 0.007 | 0.014 | 0.013 |
| CCI | 96.000 | 95.600 | 0.000 | 0.000 | 95.400 | 94.000 | 94.000 | 93.400 | 95.600 |
| Time(s) | 0.036 | 0.040 | 0.034 | 0.037 | 0.005 | 0.076 | 0.088 | 15.787 | 157.250 |
| **Scenario 1.a: 5U (C,U) ∼ Gaussian** | | | | | | | | | |
| Bias | -0.003 | -0.004 | 0.347 | 0.347 | -0.011 | 0.002 | 0.000 | 0.016 | 0.014 |
| ASE | 0.065 | 0.062 | 0.047 | 0.045 | 0.206 | 0.131 | 0.130 | 0.167 | 0.163 |
| ESE | 0.059 | 0.057 | 0.046 | 0.044 | 0.205 | 0.132 | 0.131 | 0.162 | 0.155 |
| MSE | 0.004 | 0.003 | 0.123 | 0.122 | 0.042 | 0.017 | 0.017 | 0.026 | 0.024 |
| CCI | 97.000 | 96.200 | 0.000 | 0.000 | 96.000 | 95.800 | 95.800 | 93.600 | 95.400 |
| Time(s) | 0.042 | 0.048 | 0.034 | 0.037 | 0.006 | 0.081 | 0.091 | 41.200 | 409.142 |
| **Scenario 1.b: 2U (C,U) ∼ non Gaussian** | | | | | | | | | |
| Bias | 0.003 | 0.002 | 0.303 | 0.302 | 0.000 | 0.004 | 0.004 | 0.001 | 0.004 |
| ASE | 0.058 | 0.055 | 0.051 | 0.048 | 0.184 | 0.072 | 0.081 | 0.121 | 0.119 |
| ESE | 0.059 | 0.056 | 0.052 | 0.050 | 0.185 | 0.092 | 0.084 | 0.110 | 0.103 |
| MSE | 0.003 | 0.003 | 0.094 | 0.094 | 0.034 | 0.009 | 0.007 | 0.012 | 0.011 |
| CCI | 93.800 | 93.800 | 0.000 | 0.000 | 96.200 | 86.600 | 93.200 | 95.200 | 96.600 |
| Time(s) | 0.043 | 0.045 | 0.034 | 0.036 | 0.006 | 0.078 | 0.097 | 10.258 | 100.303 |
| **Scenario 1.a: 5U (C,U) ∼ non Gaussian** | | | | | | | | | |
| Bias | 0.001 | 0.001 | 0.432 | 0.433 | 0.002 | -0.006 | -0.006 | -0.002 | -0.003 |
| ASE | 0.058 | 0.055 | 0.050 | 0.047 | 0.186 | 0.080 | 0.087 | 0.118 | 0.116 |
| ESE | 0.057 | 0.055 | 0.048 | 0.046 | 0.189 | 0.101 | 0.092 | 0.116 | 0.113 |
| MSE | 0.003 | 0.003 | 0.189 | 0.190 | 0.036 | 0.010 | 0.008 | 0.013 | 0.013 |
| CCI | 95.600 | 96.400 | 0.000 | 0.000 | 94.400 | 87.400 | 94.200 | 94.800 | 95.800 |
| Time(s) | 0.047 | 0.055 | 0.030 | 0.033 | 0.007 | 0.079 | 0.093 | 28.621 | 284.602 |

Abbreviations: ASE, asymptotic standard error; CCI, coverage rate of 95% confidence interval; ESE, empirical standard error; MSE, mean square error; Time(s), mean computational time in seconds; OR, odds ratio; 2*U*, two unobserved confounders; 5*U*, five unobserved confounders
