# Supplementary material for: Health administrative data enrichment using cohort information: Comparative evaluation of methods by simulation and application to real data
Source: PLoS One. 2019 Jan 31;14(1):e0211118. doi: 10.1371/journal.pone.0211118 (PMC6354983; doi:10.1371/journal.pone.0211118)
Supplement: S2 Table — (DOCX) [file pone.0211118.s003.docx]

**S2 Table. Simulation results for the estimate of β=log(OR_YX_ ) when the external validation sample is external and not representative because the inclusion probability depends on X, Y, C, C ∗ Y (Scenario 2.a)**

|  | **UC_MAIN** | **UC_POOL** | **C_MAIN** | **C_POOL** | **UC_VAL** | **TSC** | **TSC_SP** | **MICE10** |
| --- | --- | --- | --- | --- | --- | --- | --- | --- |
| **logit(P(M = 1)) = −2.7 + log(4)X** | | | | | | | | |
| Bias | 0.001 | 0.001 | -0.310 | -0.310 | -0.001 | 0.004 | 0.004 | 0.006 |
| ASE | 0.054 | 0.050 | 0.048 | 0.045 | 0.161 | 0.076 | 0.077 | 0.122 |
| ESE | 0.054 | 0.050 | 0.047 | 0.044 | 0.157 | 0.083 | 0.083 | 0.126 |
| MSE | 0.003 | 0.002 | 0.098 | 0.098 | 0.024 | 0.007 | 0.007 | 0.016 |
| CCI | 94.800 | 96.200 | 0.000 | 0.000 | 95.800 | 92.200 | 92.400 | 93.200 |
| Time(s) | 0.040 | 0.043 | 0.036 | 0.040 | 0.006 | 0.082 | 0.101 | 16.316 |
| **logit(P(M = 1)) = −2.7 + log(4)Y** | | | | | | | | |
| Bias | 0.002 | 0.002 | -0.308 | -0.308 | 0.008 | 0.004 | 0.004 | 0.009 |
| ASE | 0.054 | 0.050 | 0.048 | 0.045 | 0.160 | 0.075 | 0.075 | 0.121 |
| ESE | 0.052 | 0.049 | 0.048 | 0.045 | 0.162 | 0.078 | 0.078 | 0.123 |
| MSE | 0.003 | 0.002 | 0.097 | 0.097 | 0.026 | 0.006 | 0.006 | 0.015 |
| CCI | 95.600 | 96.600 | 0.000 | 0.000 | 94.800 | 93.600 | 94.000 | 93.800 |
| Time(s) | 0.042 | 0.044 | 0.043 | 0.045 | 0.006 | 0.094 | 0.123 | 16.408 |
| **logit(P(M = 1)) = −2.7 + log(4)C_2_** | | | | | | | | |
| Bias | 0.000 | 0.000 | -0.312 | -0.312 | -0.001 | 0.001 | 0.001 | 0.004 |
| ASE | 0.054 | 0.051 | 0.048 | 0.046 | 0.160 | 0.075 | 0.075 | 0.123 |
| ESE | 0.054 | 0.050 | 0.048 | 0.046 | 0.154 | 0.077 | 0.078 | 0.115 |
| MSE | 0.003 | 0.003 | 0.100 | 0.099 | 0.024 | 0.006 | 0.006 | 0.013 |
| CCI | 94.200 | 94.200 | 0.000 | 0.000 | 96.000 | 94.800 | 94.800 | 94.600 |
| Time(s) | 0.045 | 0.049 | 0.044 | 0.050 | 0.006 | 0.098 | 0.117 | 16.824 |
| **logit(P(M = 1)) = −2.5 + log(2)C_1_ + log(2)Y + log(4)C_1_ ∗ Y** | | | | | | | | |
| Bias | -0.002 | -0.001 | -0.313 | -0.312 | 0.007 | 0.005 | 0.004 | 0.010 |
| ASE | 0.054 | 0.050 | 0.048 | 0.045 | 0.158 | 0.078 | 0.079 | 0.124 |
| ESE | 0.053 | 0.050 | 0.047 | 0.044 | 0.159 | 0.079 | 0.079 | 0.129 |
| MSE | 0.003 | 0.002 | 0.100 | 0.099 | 0.025 | 0.006 | 0.006 | 0.017 |
| CCI | 95.800 | 95.800 | 0.000 | 0.000 | 94.600 | 95.400 | 95.200 | 92.800 |
| Time(s) | 0.045 | 0.051 | 0.047 | 0.048 | 0.005 | 0.103 | 0.129 | 17.789 |

Abbreviations: ASE, asymptotic standard error; CCI, coverage rate of 95% confidence interval; ESE, empirical standard error; MSE, mean square error; OR, odds ratio; P(M = 1), Probability of belonging to the validation data; Time(s), mean computational time in seconds
