# Supplementary material for: Health administrative data enrichment using cohort information: Comparative evaluation of methods by simulation and application to real data
Source: PLoS One. 2019 Jan 31;14(1):e0211118. doi: 10.1371/journal.pone.0211118 (PMC6354983; doi:10.1371/journal.pone.0211118)
Supplement: S3 Table — (DOCX) [file pone.0211118.s004.docx]

**S3 Table. Simulation results for the estimate of β = log(OR_YX_) when the validation sample is external and not representative because the inclusion probability depends on X+Y or X*Y (Scenario 2.b)**

|  | **UC_MAIN** | **UC_POOL** | **C_MAIN** | **C_POOL** | **UC_VAL** | **TSC** | **TSC_SP** | **MICE10** |
| --- | --- | --- | --- | --- | --- | --- | --- | --- |
| **logit(P(M = 1)) = −2.6 + log(2)X + log(2)Y** | | | | | | | | |
| Bias | -0.001 | 0.027 | -0.311 | -0.283 | -0.061 | 0.030 | 0.030 | 0.026 |
| ASE | 0.054 | 0.050 | 0.048 | 0.045 | 0.148 | 0.073 | 0.073 | 0.113 |
| ESE | 0.055 | 0.051 | 0.048 | 0.045 | 0.143 | 0.078 | 0.078 | 0.109 |
| MSE | 0.003 | 0.003 | 0.099 | 0.082 | 0.024 | 0.007 | 0.007 | 0.012 |
| CCI | 94.600 | 90.000 | 0.000 | 0.000 | 93.400 | 92.200 | 92.200 | 95.600 |
| Time(s) | 0.042 | 0.051 | 0.044 | 0.049 | 0.006 | 0.096 | 0.119 | 16.639 |
| **logit(P(M = 1)) = −3.2 + log(4)X + log(4)Y** | | | | | | | | |
| Bias | 0.001 | 0.099 | -0.310 | -0.211 | -0.239 | 0.119 | 0.120 | 0.100 |
| ASE | 0.054 | 0.049 | 0.048 | 0.044 | 0.150 | 0.072 | 0.072 | 0.120 |
| ESE | 0.054 | 0.048 | 0.049 | 0.044 | 0.139 | 0.074 | 0.074 | 0.111 |
| MSE | 0.003 | 0.012 | 0.098 | 0.046 | 0.077 | 0.020 | 0.020 | 0.022 |
| CCI | 95.400 | 47.800 | 0.000 | 0.200 | 64.600 | 63.200 | 64.000 | 84.400 |
| Time(s) | 0.056 | 0.051 | 0.040 | 0.043 | 0.006 | 0.094 | 0.137 | 16.401 |
| **logit(P(M = 1)) = −2.7 + log(2)X + log(2)Y + log(2)X∗ Y** | | | | | | | | |
| Bias | -0.003 | 0.122 | -0.314 | -0.188 | 0.456 | 0.076 | 0.076 | 0.129 |
| ASE | 0.054 | 0.049 | 0.048 | 0.044 | 0.147 | 0.072 | 0.072 | 0.113 |
| ESE | 0.055 | 0.049 | 0.050 | 0.044 | 0.144 | 0.080 | 0.080 | 0.112 |
| MSE | 0.003 | 0.017 | 0.101 | 0.037 | 0.229 | 0.012 | 0.012 | 0.029 |
| CCI | 94.200 | 28.800 | 0.000 | 1.800 | 12.400 | 79.800 | 80.400 | 78.200 |
| Time(s) | 0.048 | 0.051 | 0.044 | 0.053 | 0.006 | 0.096 | 0.132 | 17.521 |
| **logit(P(M = 1)) = −2.5 + log(2)X + log(2)Y − log(2)X∗ Y** | | | | | | | | |
| Bias | -0.004 | -0.058 | -0.315 | -0.368 | -0.631 | -0.013 | -0.013 | -0.057 |
| ASE | 0.054 | 0.051 | 0.048 | 0.046 | 0.158 | 0.073 | 0.073 | 0.116 |
| ESE | 0.053 | 0.050 | 0.047 | 0.045 | 0.152 | 0.076 | 0.077 | 0.119 |
| MSE | 0.003 | 0.006 | 0.101 | 0.137 | 0.421 | 0.006 | 0.006 | 0.017 |
| CCI | 95.600 | 80.200 | 0.000 | 0.000 | 1.200 | 93.400 | 93.200 | 90.400 |
| Time(s) | 0.051 | 0.063 | 0.044 | 0.045 | 0.007 | 0.094 | 0.132 | 17.069 |

Abbreviations: ASE, asymptotic standard error; CCI, coverage rate of 95% confidence interval; ESE, empirical standard error; MSE, mean square error; OR, odds ratio; P(M = 1), Probability of belonging to the validation data; Time(s), mean computational time in seconds
