# Supplementary material for: Health administrative data enrichment using cohort information: Comparative evaluation of methods by simulation and application to real data
Source: PLoS One. 2019 Jan 31;14(1):e0211118. doi: 10.1371/journal.pone.0211118 (PMC6354983; doi:10.1371/journal.pone.0211118)
Supplement: S4 Table — (DOCX) [file pone.0211118.s005.docx]

**S4 Table. Simulation results for the estimate of β = log(OR_YX_) when the validation sample is internal and not representative because the inclusion probability depends on X *Y (Scenario 3)**

|  | **UC_MAIN** | **C_MAIN** | **UC**_**VAL** | **TSC** | **TSC_SP** | **MICE10** |
| --- | --- | --- | --- | --- | --- | --- |
| **logit(P(M = 1)) = −2.7 + log(2)X + log(2)Y + log(2)X ∗ Y** | | | | | | |
| Bias | -0.001 | -0.310 | 0.451 | -0.047 | -0.046 | 0.001 |
| ASE | 0.051 | 0.046 | 0.132 | 0.068 | 0.069 | 0.106 |
| ESE | 0.048 | 0.043 | 0.128 | 0.069 | 0.069 | 0.096 |
| MSE | 0.002 | 0.098 | 0.220 | 0.007 | 0.007 | 0.009 |
| CCI | 96.600 | 0.000 | 7.200 | 90.200 | 90.200 | 94.600 |
| Time(s) | 0.058 | 0.051 | 0.009 | 0.101 | 0.122 | 20.469 |
| **logit(P(M = 1)) = −2.5 + log(2)X + log(2)Y − log(2)X ∗ Y** | | | | | | |
| Bias | 0.005 | -0.307 | -0.628 | 0.048 | 0.049 | 0.001 |
| ASE | 0.051 | 0.046 | 0.142 | 0.069 | 0.069 | 0.104 |
| ESE | 0.048 | 0.044 | 0.143 | 0.069 | 0.069 | 0.103 |
| MSE | 0.002 | 0.096 | 0.414 | 0.007 | 0.007 | 0.011 |
| CCI | 96.200 | 0.000 | 1.000 | 90.800 | 90.600 | 95.200 |
| Time(s) | 0.049 | 0.042 | 0.006 | 0.087 | 0.111 | 20.117 |
| **logit(P(M = 1)) = −2.8 + log(2)X + log(2)Y + log(4)X ∗ Y** | | | | | | |
| Bias | 0.002 | -0.308 | 0.878 | -0.084 | -0.084 | 0.004 |
| ASE | 0.051 | 0.046 | 0.133 | 0.068 | 0.068 | 0.112 |
| ESE | 0.051 | 0.046 | 0.132 | 0.075 | 0.075 | 0.111 |
| MSE | 0.003 | 0.097 | 0.788 | 0.013 | 0.013 | 0.012 |
| CCI | 94.400 | 0.000 | 0.000 | 72.600 | 72.800 | 93.600 |
| Time(s) | 0.060 | 0.053 | 0.008 | 0.093 | 0.123 | 20.830 |
| **logit(P(M = 1)) = −2.4 + log(2)X + log(2)Y − log(4)X ∗ Y** | | | | | | |
| Bias | -0.002 | -0.312 | -1.246 | 0.074 | 0.076 | -0.002 |
| ASE | 0.051 | 0.046 | 0.158 | 0.069 | 0.070 | 0.101 |
| ESE | 0.049 | 0.043 | 0.159 | 0.071 | 0.072 | 0.101 |
| MSE | 0.002 | 0.100 | 1.578 | 0.010 | 0.011 | 0.010 |
| CCI | 95.200 | 0.000 | 0.000 | 82.400 | 81.600 | 94.200 |
| Time(s) | 0.050 | 0.046 | 0.007 | 0.092 | 0.119 | 20.913 |

Abbreviations: ASE, asymptotic standard error; CCI, coverage rate of 95% confidence interval; ESE, empirical standard error; MSE, mean square error; OR, odds ratio; P(M = 1), Probability of belonging to the validation data; Time(s), mean computational time in seconds
