# Supplementary material for: Health administrative data enrichment using cohort information: Comparative evaluation of methods by simulation and application to real data
Source: PLoS One. 2019 Jan 31;14(1):e0211118. doi: 10.1371/journal.pone.0211118 (PMC6354983; doi:10.1371/journal.pone.0211118)
Supplement: S5 Table — (DOCX) [file pone.0211118.s006.docx]

**S5 Table. Simulation results for the estimate of β = log(OR_YX_) when the validation sample is external and not representative because the inclusion probability depends on U (Scenario 2.c)**

|  | | **UC_MAIN** | **UC_POOL** | **C_MAIN** | **C_POOL** | **UC_VAL** | **TSC** | **TSC_SP** | **MICE10** |
| --- | --- | --- | --- | --- | --- | --- | --- | --- | --- |
| **logit(P(M = 1)) = −2.2 + log(2)U_1_** | | | | | | | | | |
| Bias | -0.001 | | -0.001 | -0.313 | -0.320 | 0.002 | -0.025 | -0.025 | -0.015 |
| ASE | 0.054 | | 0.051 | 0.048 | 0.046 | 0.173 | 0.076 | 0.076 | 0.124 |
| ESE | 0.054 | | 0.052 | 0.048 | 0.046 | 0.181 | 0.085 | 0.086 | 0.122 |
| MSE | 0.003 | | 0.003 | 0.100 | 0.105 | 0.033 | 0.008 | 0.008 | 0.015 |
| CCI | 95.200 | | 94.800 | 0.000 | 0.000 | 94.000 | 90.400 | 90.600 | 93.600 |
| Time(s) | 0.046 | | 0.057 | 0.050 | 0.051 | 0.006 | 0.103 | 0.125 | 17.903 |
| **logit(P(M = 1)) = −2.7 + log(4)U_1_** | | | | | | | | | |
| Bias | | -0.001 | -0.001 | -0.311 | -0.334 | -0.002 | -0.096 | -0.096 | -0.091 |
| ASE | | 0.054 | 0.051 | 0.048 | 0.046 | 0.180 | 0.074 | 0.074 | 0.127 |
| ESE | | 0.057 | 0.054 | 0.052 | 0.049 | 0.182 | 0.085 | 0.085 | 0.132 |
| MSE | | 0.003 | 0.003 | 0.099 | 0.114 | 0.033 | 0.016 | 0.017 | 0.026 |
| CCI | | 95.000 | 94.600 | 0.000 | 0.000 | 94.600 | 69.800 | 69.200 | 83.000 |
| Time(s) | | 0.039 | 0.043 | 0.036 | 0.040 | 0.005 | 0.081 | 0.103 | 16.454 |
| **logit(P(M = 1)) = −2.3 + log(2)U_1_ + log(2)X∗ U_1_** | | | | | | | | | |
| Bias | | 0.000 | -0.001 | -0.311 | -0.291 | -0.004 | -0.262 | -0.262 | -0.271 |
| ASE | | 0.053 | 0.051 | 0.048 | 0.046 | 0.159 | 0.058 | 0.058 | 0.101 |
| ESE | | 0.053 | 0.050 | 0.047 | 0.045 | 0.153 | 0.069 | 0.070 | 0.093 |
| MSE | | 0.003 | 0.002 | 0.099 | 0.087 | 0.023 | 0.074 | 0.074 | 0.082 |
| CCI | | 95.600 | 95.600 | 0.000 | 0.000 | 95.800 | 1.800 | 1.800 | 23.600 |
| Time(s) | | 0.051 | 0.054 | 0.050 | 0.051 | 0.007 | 0.107 | 0.139 | 19.314 |
| **logit(P(M = 1)) = −2.5 + log(2)U_1_ + log(2)Y∗ U_1_** | | | | | | | | | |
| Bias | | -0.002 | 0.000 | -0.311 | -0.360 | -0.029 | 0.143 | 0.143 | 0.134 |
| ASE | | 0.054 | 0.051 | 0.048 | 0.046 | 0.185 | 0.087 | 0.086 | 0.167 |
| ESE | | 0.051 | 0.048 | 0.047 | 0.044 | 0.177 | 0.100 | 0.100 | 0.163 |
| MSE | | 0.003 | 0.002 | 0.099 | 0.131 | 0.032 | 0.030 | 0.030 | 0.045 |
| CCI | | 97.200 | 97.600 | 0.000 | 0.000 | 96.200 | 64.800 | 63.600 | 87.600 |
| Time(s) | | 0.039 | 0.043 | 0.036 | 0.040 | 0.006 | 0.084 | 0.102 | 16.301 |

Abbreviations: ASE, asymptotic standard error; CCI, coverage rate of 95% confidence interval; ESE, empirical standard error; MSE, mean square error; OR, odds ratio; P(M = 1), Probability of belonging to the validation data; Time(s), mean computational time in seconds
